# State-of-the-Art Age Determination Methods for Amphibians and Reptiles

**DOI:** 10.3390/ani15182722

**Published:** 2025-09-17

**Authors:** Fabio Maria Guarino, Marcello Mezzasalma

**Affiliations:** 1 Department of Biology, University of Naples Federico II, Via Cinthia 26, 80126 Naples, Italy; 2 Department of Biology, Ecology and Earth Science, University of Calabria, Via P. Bucci 4/B, 87036 Rende, Italy; marcello.mezzasalma@unical.it

**Keywords:** amphibians, reptiles, age determination methods, accuracy, range of applicability

## Abstract

In this work, we provide an overview of the age determination methods of amphibians and reptiles, critically evaluating their methodological principles, utility (including range of applicability), as well as experimental and biological limitations. Potentially, the most reliable age estimation method is the capture–mark–recapture (CMR) technique, allowing a precise chronological age estimation of wild individuals. However, this is a time-consuming method and is not always applicable. Among the indirect methods of age estimation, skeletochronology is one of the most reliable techniques and has been successfully applied in numerous species of amphibians and reptiles. Skeletochronology is based on the interpretation of the growth marks (especially lines of arrested growth or LAGs) that form in hard-mineralized tissues. Other indirect methods such as the counting of growth marks on epidermal scutes, dermal scales, and claw sections have shown to be less reliable and/or have a more limited range of applicability. Recently, the estimation of chronological age using molecular methods has acquired new perspectives from different approaches. Among them, measurements of telomere length and DNA methylation represent promising and minimally invasive methods, but their effective use requires further refinement and testing on a larger number of species.

## Simple Summary

Accurately assessing animals’ age parameters (age at maturity, mean age, and longevity) and growth-related parameters is fundamental in many fields of biology. Numerous methods with varying degrees of accuracy and precision have been developed to estimate the age and growth rates of amphibians and reptiles in the wild. The capture–mark–recapture technique potentially provides the most reliable data on age–size relationships, but requires a large amount of work, is time-consuming, and is often impractical for some wild populations. Skeletochronology has proven to be the most reliable among indirect age determination methods and has been successfully applied in numerous species of amphibians and reptiles. Skeletochronology is based on the interpretation of the growth marks that form within mineralized tissues, such as bones, during animals’ lives. Other indirect methods of age estimation based on the same principle (counting of growth marks), but applied to hard non-skeletal structures (epidermal scutes, claws, osteoderms), result in less reliable assessments and/or cannot be applied to all taxa. Recently, the use of molecular and biochemical methods such as measurements of telomere length and DNA methylation has provided new promising tools for the estimation of age, but these methods still need further refinement and testing on a larger number of species.

## 1. Introduction

Understanding the timing and evolution of life histories is a key theme in evolutionary biology as well as crucial for studies in ecology, zoology, and conservation biology [1,2,3,4,5,6]. Age at maturity, mean age, longevity, and growth-related parameters are among the most important organismal traits and allow predictions about species’ life histories. Amphibians and reptiles represent two particularly interesting groups to better understand the proximate and evolutionary causes of aging and longevity in vertebrates. While they constitute two very different groups of vertebrates in terms of evolutionary history and biological characteristics, they can be considered together in terms of addressing age estimation methods because many of these methods can be applied to either group.

Amphibians and reptiles include many species with a maximum longevity of higher than 100 years (e.g., *Proteus salamanders*, *Galapagos tortoise*) [1,7] as well as the current record holder for the shortest life span in tetrapods (about five months, in the Labord’s chameleon, *Furcifer labordi*) [8]. Wild amphibians and reptiles show a greater variability in growth rates and longevity than birds and mammals [5]. Furthermore, in amphibians and reptiles the age structure and other demographic traits can highly differ among populations of the same species living at different altitudes and/or latitudes (i.e., [9,10,11,12,13,14]). Like endothermic vertebrates, in amphibians and reptiles both growth and longevity rates are correlated to the age of sexual maturity [5].

Multiple factors can exert different selective pressures that affect the longevity of amphibian and reptile species. Species exposed to reduced extrinsic and intrinsic mortality pressures (less predation, lower metabolic rates, and shorter activity periods) live longer [15,16]. However, it should be taken into account that in longevity studies, the chances of finding older individuals increase with a larger study sample.

In general, for animals, we can distinguish life span from life expectancy [17]. The first (also referred to as “maximum”, “potential”, or “specific longevity”) represents the maximum age that the species can attain in optimal conditions. The life span is generally fixed, although in various animal species can be variable to some extent and, in some cases, it can be extended by the assumption of some particular substances (i.e., lithium, see [18,19]). In turn, life expectancy (also termed “ecological longevity”) represents the average length of the life of individuals living in the wild. It is highly dependent on environmental conditions (such as temperature, trophic resources, predation, and diseases) and it may vary among different populations. In amphibians and reptiles, the difference between life span and life expectancy can be significant, and it is usually greater than that of similar-sized endotherms. In addition, longevity in captive individuals is usually much higher than longevity in the wild for a variety of factors, including the lack of natural predators. For example, the common toad *Bufo bufo* can attain 36 years in captivity [20], whereas its longevity in the wild varies considerably among populations, but has been never documented to exceed 15 years [21]. Similarly, the longevity of the Mediterranean gecko *Hemidactylus turcicus* is 8 years in captivity while it is 4 years in the wild [22].

In general, age estimates can be defined as “absolute age” (termed also “chronological age”) when the age is estimated according to standard time measurements (usually years for long-lived species) or “relative age” (termed also “categorical age”) when the animal is assigned to an age class [23]. Relative age is also synonymous of “biological age” since it expresses the aging phase of an individual (e.g., newborn, subadult, adult) and can be defined on the basis of morpho-functional characteristics associated with individual development (e.g., skeletal changes, ontogenetic color and/or pattern changes, sexual maturity). In some field of studies, such as demography (i.e., [24,25,26,27,28]) it is necessary to infer the absolute age, while for others, including, for example, reproductive biology (i.e., [29]) or ethology (i.e., [30]), an assessment of the relative age can be appropriate for most studies.

Numerous methods with varying degrees of accuracy and precision have been developed to estimate the individual age and growth rates of amphibians and reptiles. In this contribution, we summarize and critically evaluate the methodological principle, the utility (including accuracy, efficiency, and range of applicability) as well as the experimental and biological limitations of these methods. Finally, we consider the still unresolved critical issues presented by the available methods and topics insufficiently explored, proposing some future research avenues. This review is intended as an informative introduction to age estimation for non-specialists as well as a methodological summary and a reference guide for more experienced researchers.

## 2. Materials and Methods

Peer-reviewed scientific papers were identified and selected by electronic literature searches in several engines including Web of Science^®^, PubMed^®^, Google Scholar^®^ and JSTOR^®^ from April 2025 to June 2025. We specifically searched for terms: “age estimation” or “life expectancy” or “longevity” or “lifespan” or “capture-mark-recapture” or “sclerochronology” or “skeletochronology” or “size frequency” or “growth marks” or “telomere length” or “DNA methylation” or “DNAm” or “amino acid racemization” or “bomb radiocarbon” AND “amphibians” or “reptiles” or “frogs” or “caecilians” or “salamanders” or “newts” or “amphisbaenians” or “lizards” or “snakes” or “squamates” or “turtles” or “crocodiles” or “*Sphenodon*” or “tuatara”. A further search was performed checking the literature cited by the papers retrieved during the initial search and the authors’ personal databases.

Bone terminology and relative abbreviations used in the following sections generally follow refs. [17,20].

## 3. Methods of Age Estimation

### 3.1. Capture–Mark–Recapture Method

Potentially, the most reliable age estimation method is the capture–mark–recapture (CMR) technique, which virtually allows a precise chronological age estimation of wild animals [31]. This method requires the capture of animals of a known age (preferably hatchlings and neonates) during an initial sampling period, their marking, release and recapture at periodic intervals. This technique is generally used in combination with body length measurements to infer age-specific growth rates [31,32]. Furthermore, the CMR technique can be useful for validating other methods of age estimation such as those based on the interpretation of incremental growth layers [32,33,34,35]. Besides being one of the first methods of age determination, the CMR method is also one of the most frequently used (i.e., [36,37]); for other references see [31]). Furthermore, CMR can be applied to certain taxa, such as snakes (i.e., [38,39]) for which other methods of age determination cannot be applied without sacrificing the individual or causing significant impairment (as in the case of skeletochronology, see below). The main limitations of the CMR method include requirements of long-term observations, which can hardly be achieved on some wild populations.

### 3.2. Size Frequency Method

The extrapolation from size frequency data is an indirect method for estimating age, and, in the past, it was frequently applied to amphibians [40,41] and reptiles (i.e., [42,43,44]). The main weakness of this method is that it assumes that body length and age are positively correlated, while this has been shown to be false for many species (in one or both sexes) or between different populations of the same species [24,31,45,46]. As a result, individuals that fall into the same size class can often have very different ages as shown using other methods (e.g., skeletochronology, see below). Furthermore, several studies provided evidence that linear growth may stop well before senescence in reptiles including those taxa until recently considered indeterminate growers, such as crocodilians [47,48,49,50], and/or very long-living species, such as the Blanding’s turtle *Emydoidea blandingii* [51].

### 3.3. Sclerocronology

Most of the indirect methods of age estimation are based on the correlation between the growth of individual and the progressive layering of its hard tissues such as otoliths (only for bony fishes, see [33]); epidermal scutes (i.e., [52]), claws (i.e., [53,54]), bones (i.e., [55,56,57,58,59,60,61,62,63,64,65,66,67,68,69]), teeth (only for mammals), osteoderms (i.e., [70]), and dermal scales (i.e., [71]). These methods are overall called sclerochronology, and more specifically skeletochronology when they involve only mineralized hard tissues [17,33,72] (Table 1).

While the counting of growth marks on otoliths (also termed otolithometry) has been widely applied in bony fishes (teleosts) (see [73]), it has never been used in amphibians and reptiles. In fact, unlike the otoliths of most bony fish, which are stony masses, the inner ear labyrinth of amphibians and reptiles contains much smaller crystals of calcium carbonate (otoconia or statoconia), which are unsuitable for otolithometric applications [74].

**Figure 1:**
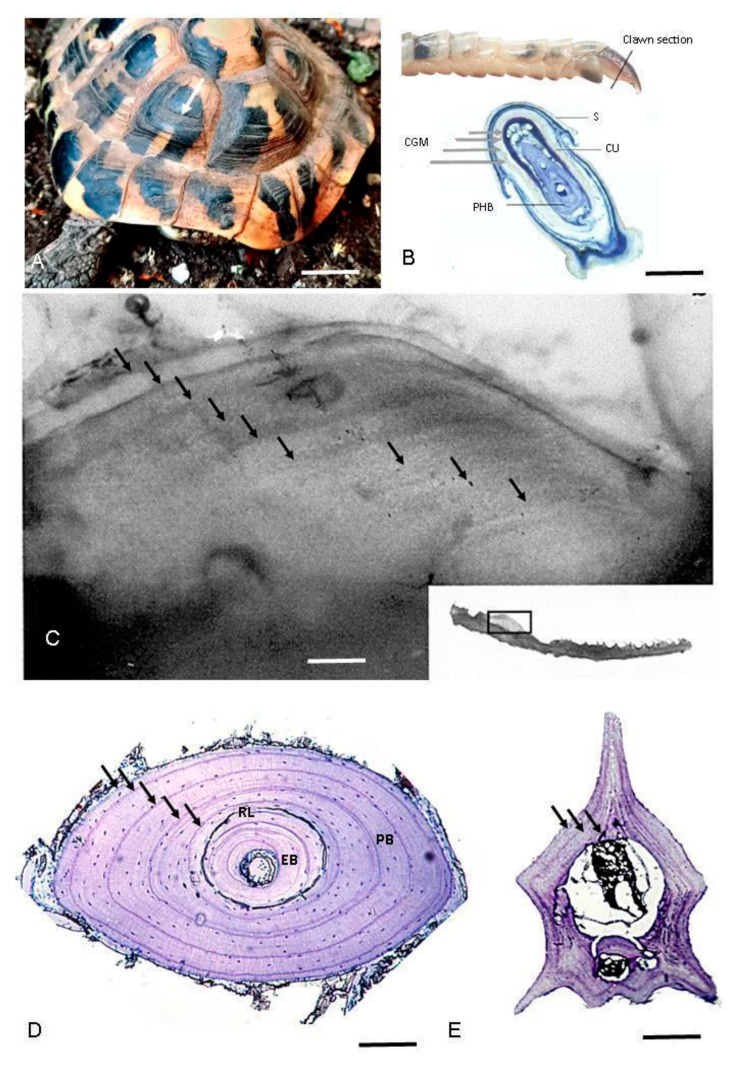
Representative growth marks in hard tissues of amphibians and reptiles. (**A**) Scute rings in *Testudo hermanni*; (**B**) Claw section of *Darevskia portschinskii* (Squamata, Lacertidae) (modified from [51]); (**C**) Growth marks seen by transparency (backlighting of the bone) in the mandibular plate from *Hierophis viridiflavus* (Squamata, Colubridae). (**D**) Phalanx cross-section of *Boehmantis microtympanum* (Anura, Mantellidae), decalcified with 5% acid nitric and stained with Mayer’s Hematoxylin; (**E**) Caudal vertebra cross-section of *Hierophis viridiflavus* (Squamata, Colubridae), decalcified with 5% acid nitric and stained with Mayer’s Hematoxylin. White arrow points to scute ring (only the first one is indicated); gray arrows point to claw growth marks; black arrows point to LAGs in the bone section. CGM: claw growth marks; EB: endosteal bone; PB, periosteal bone; PHB: phalanx bone; RL: reversal line; S: scale; and U: unguis. Scale bar corresponds to 18 mm (**A**), 200 µm (**B**), 380 µm (**C**), 130 µm (**D**), and 600 µm (**E**).

The counting of growth marks or rings on scutes (Figure 1A) has been tested only in several species of tortoises and terrapins [75,76,77,78,79,80,81]. Indeed, it finds its main constraint in the fact that there are few species of amphibians and reptiles with keratinized epidermal scutes. Moreover, according to several researchers, the use of scute rings may produce age estimates in chelonians that are, at best, approximations, except in the youngest specimens [78], in individuals near the age of maturity [82], or up to a certain age (for example 15 years in the *Gopherus polyfemus*) [79]. Furthermore, this method might be practicable for some populations, but only after calibrating the relationship between ring counts and age for each circumstance (including species and geographical location) [83]. Nevertheless, a recent long-term CMR study on *Testudo graeca* showed that, when correctly applied, individual age calculated by counting shell growth rings can be more precise than other techniques [81], underlining that while age estimation in turtles is challenging, a combination of methods could improve its accuracy.

The analysis of claw sections (Figure 1B) for the purpose of age determination could potentially be applied to a large number of reptiles but, so far, it was only performed on a few species, including the terrapins *Chrysemys picta* and *Deirochelis reticularia* [53] and some lizard species of the genus *Darevskia* [54]. In terrapins, this method was applied to individuals under 30 years of age, so there may be upper age limits on the usefulness of claw sections as a precise tool for age determination [53].

Unlike all the previously mentioned indirect methods, skeletochronological studies have been successfully applied to a large number of species of amphibians and reptiles and include several hundreds of published papers (at least 369 for amphibians, see [84], and at least 468 papers covering 236 reptile species, see [85]), representing the most used age estimation method for these vertebrates. Skeletochronology is a histological method based on the interpretation of the growth marks occurring in the mineralized tissues of an individual throughout its life cycle, corresponding to the slowing or stopping of the somatic growth, and reflecting endogenous biological rhythms, which are generally synchronized with environmental conditions [33]. In general, three categories of growth marks can be recognized in the bone: lines of arrested growth (LAGs), representing a temporary arrest of the bone growth; annuli, which corresponds to periods of slow bone deposition; zones or opaque layers, which are the widest marks and correspond to periods of active osteogenesis [17,33]. Also, one LAG can be present within an annulus. Once LAGs with annual periodicity have been identified, the age estimation of the individual can be assessed from their count (including any totally reabsorbed LAG) while growth rates can be estimated from the distance between the LAGs. In amphibians and reptiles, annual growth marks can be observed in several bones, including skull bones (i.e., [55,58,65]), vertebrae (i.e., [66,67,69]), and long, tubular bones, such as the femur, humerus, and phalanx (i.e., [60,86,87,88,89,90]). Growth marks can be detectable also in osteoderms of some reptile groups, such as crocodilians and Anguidae, as well as in the dermal scales of amphibians of the order Gymnophiona. However, within these taxa, a limited number of species (including the *Crocodylus niloticus*, [91]; *C. johnstoni* [70], *A. veronensis* [68], and *Dermophis mexicanus* [71]) have been studied in order to determine individual age through the growth mark-counting of mineralized dermal structures, with contrasting results in terms of efficiency and accuracy. In Diploglossidae, it has been shown that regenerated and non-regenerated tail osteoderms probably do not follow the same growth rates and that skeletochronology should be applied only to non-regenerated osteoderms [92]. Therefore, estimating age by counting growth marks that form in this mineralized tissue should be viewed with caution in the case of some species, i.e., crocodiles [70], and it is probably unreliable in other species [66,68,71]. The teeth are also unsuitable for skeletochronological analysis because majority of amphibians and reptiles are polyphyodonts [93,94]. In a few exceptions, such as the agamids where post-canine acrodont teeth are not replaced during their lifetime, the growth layers formed in the teeth are indistinct when they are examined in section and cannot be used for age determination [95]. On the other hand, the use of so-called tide marks on tooth roots, which are visible, for example, in the *Crocodylus niloticus*, is currently anecdotal [72]. However, available data show that, while growth marks can be visualized in very thin flat bones by backlighting them (Figure 1C), they have better optical sharpness using stained decalcified bone sections [72] (Figure 1D,E). In general, long bones are the most useful and reliable skeletal elements with regard to growth mark visualization and ease in sample preparation [14,33,96]. Furthermore, long bones such as the femur, humerus, and phalanges usually provide corresponding age estimations in amphibians and reptiles [20,63,97]. A few exceptions are represented by some very long-living species (e.g., *Sphenodon punctatus*, *Bombina variegata*, and *Homonota darwini*) in which age assessments based on the phalanges are slightly underestimated compared with those based on the femur [64,98,99]. Furthermore, since different bones can differ in growth patterns and in bone remodeling rates [20,100,101], it can be useful to perform preliminary comparisons between the different types of long bones in understudied taxa, before undertaking a skeletochronological study. Obviously, limbless taxa, such as Gymnophiona, snakes, and many lizard species (i.e., Dibamidae, Anguidae) or taxa with much reduced limbs (i.e., several species of skinks) represent a special challenge. In these taxa, skeletochronology can be applied to skull bones, such as the ectopterygoid (as in the snake *Trimesurus flavoviridis*) [102], the supra-angular of the mandibula (as in the snakes *Vipera aspis* and *Trimesurus flavoviridis*) [58,102,103] or the caudal vertebrae (as in the cecilian *Geotrypetes seraphini*, in the limbless lizards of the genus *Anguis*, and in the snakes *Thamnophis* and *Trimesurus*) [66,68,69,104,105]. However, excluding studies on samples from museum collections (i.e., [106,107,108], skeletochronology performed on cranial bones, vertebrae, femur or humerus usually requires the sacrifice of the animal. On the contrary, skeletochronology performed on phalanges obtained by toe-clipping allows both the marking of individuals and the collection of bone samples for skeletochronological analysis without the sacrifice of any animal, potentially also enabling demographic studies over long intervals of time [14,28,109]. Technical accounts of skeletochronology including its validation and possible solutions to the main difficulties (e.g., endosteal resorption of LAGs, double LAGs, supernumerary LAGs) encountered in skeletochronological studies are given by [14,33,110,111]. It is worth mentioning that the study of Schucht et al. [112] questioned the ambiguity of histology-based skeletochronology because halved bones (at level of the area with the smallest medullary cavity) provided some discrepant age estimates when comparing petrographic ground sections and conventional microtomized sections. Taking into account the fundamental differences between these two histological techniques, more research is needed to better standardize some methodological aspects, with particular regard to the unambiguous recognition of the different types of growth marks.

### 3.4. Other Morphological Methods

Among the traditional morphological methods of age estimation (other than skeletochronology) is the measurement of the eyeball lens, which grows continually, though not continuously, throughout the animal’s life. Although this method has been widely tested for mammals, very limited data are available for amphibians and reptiles (for a list of species see [73,77,113]. Furthermore, as pointed out by Zug [77], small differences in the lens mass can lead to disproportionate differences in age estimation; consequently, the protocol (including the type of fixative and duration of lens fixation) should be standardized as much as possible.

Some other morphological methods of age determination have been proposed in the past but later proven ineffective. One of the best-known examples is the count of testicular lobes in salamanders, which were thought to form progressively over successive breeding seasons and could therefore be used to estimate individual age (i.e., [114,115]). However, in a study on the *Lissotriton vulgaris* and *T. cristatus,* a considerable variation in lobe numbers within age classes (determined by skeletochronology) was found and no male showed more than three or four lobes [116]. The alleged correlation between lobe number and individual age was later refuted [117], and the method has since fallen out of use.

### 3.5. Molecular Methods

In recent years, the estimation of chronological age using molecular methods has acquired new perspectives from different approaches. In general, different molecular methods represent indirect methods of age determination, similarly to sclerochronology. After opportune calibrations (e.g., for the taxon, tissue, or cell type) they can provide either relative or absolute age estimates with a margin of error (of, e.g., 3–10 years). Telomere length (TL) shortening is a well-known hallmark of both cellular senescence and organismal aging [118]. TL shortening with body length and individual age has been tested on different taxa of amphibians and reptiles, using different molecular techniques such as Southern blot and qPCR [119,120,121,122].

The first experimental study on a natural amphibian population (of *Epidalea calamita*) reported a clear correlation between telomere shortening and age, especially during the first 1–2 years [122]. However, besides providing some interesting results, methods based on TL also showed several limitations, and there are still too few studies to conclude its full reliability in amphibians and reptiles. In particular, age determination using TL has been applied in several studies on different lizard taxa (including, e.g., the *Psammodromus algirus*, *Lacerta agilis*, *Chlamydosaurus kingii*, and *Ctenophorus pictus*), showing contrasting results [123,124,125]. Overall, these studies showed a correlation of telomere shortening with age in some taxa but also a lack of universality. In fact, TL patterns appear to vary among species, sexes, tissues, and cell types and are prone to variations linked to other factors [123,124,125]. For example, in the sand lizard (*L. agilis*), differences in telomere length were correlated to sex and stress patterns; in females, an increase in the TL was correlated with age, but in males, no distinct age pattern was recorded, and TL was negatively impacted by tail autotomy and larger body size [125].

Studies on the epigenetic process of DNA methylation (DNAm) are another recent promising experimental approach for age determination. The methodology employed is based on the quali–quantitative detection and measurement of changes in DNAm occurring in an individual during the natural course of aging. In general, the process of DNAm refers to the addition to the 5′ cytosine of cytosine–guanine pairs (CpGs) of a methyl group (CH3) by the DNA methyltransferase enzyme class as a part of mechanisms of DNA packaging and gene regulation [126].

In vertebrates, DNAm is necessary for several processes including regular development, inactivation of genes and whole chromosomes (e.g., X chromosomes in mammals), suppression of transposable elements, and genomic imprinting [127].

To date, DNAm techniques for age estimation have been used several times for mammals and tested in birds [128] and, to a limited extent, amphibians [129,130] but not in reptiles. In particular, two species of the genus *Xenopus* (*X. laevis* and *X. tropicalis*) were used as model organisms to develop age-related epigenetic clocks to estimate relative and chronological age [129,130]. Interestingly, a combination of the human and clawed frog epigenetic clock showed evolutionarily conserved methylation patterns, enabling age estimation in both species using a single formula [130].

However, similarly to TL, DNAm also presents several issues as a reliable method for age determination. In fact, the general methylation status is not fixed within any single organisms, species, or cell type. In particular, different organisms usually exhibit differential levels of DNAm in different tissues and/or age and developmental stage (i.e., [131,132], and different cell types can regulate DNAm in response to physiological and environmental changes [126]. Overall, despite presenting a high diagnostic potential, techniques of age determination-based DNAm are more expensive when compared with some alternatives and might prove challenging to standardize.

### 3.6. Biochemical Methods

Amino acid racemization (AAR) has been used in age determination in fish and mammals [73], but its reliability in amphibians and reptiles is currently unexplored and would likely require some methodological adaptations. The method is based on the evaluation of the temporal rates of conversion of L-amino acids to their D-forms over time, and it is potentially able to provide aging estimates of preserved museum samples or even fossils [133]. The efficiency of this method is linked to the identification of tissues with low-turnover rates in order to accurately measure aging [134]. In vertebrates, AAR has provided age estimates with a lower accuracy (of about +/−3–10 years) compared with other methods, and its limitations also include a potentially destructive sampling and the absence of calibration in most taxa [73,135]. Nevertheless, the potential use of AAR on preserved samples offers promising applications of the method on biological samples from public and private collections, avoiding invasive practices on endangered natural populations.

Radiocarbon methods (particularly bomb-^14^C) have been used in the age assessment of turtles [136] but not in amphibians. In particular, they have been successfully applied to living specimens and conserved hard tissues of Hawksbill sea turtles (*Eretmochelys imbricata*), helping to provide individual age estimates and date keratinized growth bands on the scute [136]. Using bomb-^14^C, Van Houtan et al. [136] found that about eight scute rings were deposited on the scute each year, aligning chronological data with biological age [136]. In the end, while radiocarbon dating had very limited empirical applications in reptiles, its use appears promising and potentially extendable to amphibian taxa.

Furthermore, techniques using stable isotopes of carbon and nitrogen have been applied to reconstruct seasonal feeding patterns and timeframes of tissue turnover in turtles and lizards (i.e., [137,138]), but they do not provide direct aging estimates. However, when isotope profiling is used in combination with skeletochronology (applied on growth lines) it may provide some useful correlations between age (counted via LAGs) and isotopic changes, helping to better understand life-stage events, feeding patterns, and habitat transitions [139].

## 4. Future Research Perspectives

Since the end of the last century, numerous studies conducted on the growth and longevity of different species of amphibians and reptiles have greatly improved our understanding of their demographic life-history traits, mainly using skeletochronology (i.e., [11,27,34,109,140,141,142]). However, some aspects remain insufficiently explored, including whether variations in age structure or in other demographic traits can be attributed to natural geographical variations, climate change, or other human-induced effects (i.e., chemical pollution, land cover change). For example, a recent study by Zamaletdinov et al. [143] on three green frog species (genus *Pelophylax*) showed specific characteristics of their age structure in relation to the degree of habitat transformation. Considering both their main limitations and exciting future perspectives, studies on age at maturity, mean age, longevity, and growth-related parameters remain fundamental windows on the natural and evolutionary history of vertebrates, which are potentially able to determine novel scenarios on the macroevolution of aging and life itself.

Although phalangeal skeletochronology can be considered one of the most efficient non-lethal methods for estimating the age and growth in most amphibians and reptiles, it presents some critical issues that can prevent an accurate LAG counting. For example, bone remodeling can totally destroy some LAGs [17,20,110,111], possible aperiodic (false) or supernumerary LAGs [33,72] can be confused with true LAGs, and very closely adjacent LAGs might be hard to correctly count in the outer margin of the bone sections of the longest-lived individuals [60]. To date, while it is possible to approximate the number of completely resorbed LAGs using the back calculation method [33,144,145,146], the false or supernumerary LAGs remain difficult to address and are often the cause of approximations in absolute age determination [112]. Therefore, the development of non-lethal age estimation methods which can be alternative to or synergic with phalangeal skeletochronology is desirable for challenging species (e.g., snakes, lizards with largely reduced limbs, or limbless taxa) and can be useful also to overcome the aforementioned general difficulties of the skeletochronological method. In particular, promising molecular and biochemical methods (with particular regard to AAR and DNAm) should be further developed and tested on a wider variety of taxa of amphibians and reptiles as they may either provide non-destructive alternatives to skeletochronology or refine the age estimation based on LAG count.

## 5. Conclusions

Studies on the age determination of individuals belonging to natural populations are fundamental to better understanding population dynamics and structure, life-history strategies, and ecological interactions. Their application ultimately informs conservation actions and policies of wildlife management. This importance is reflected in the ongoing research of accurate, efficient, and non-invasive universal methodologies, being able to cover distinct organisms with marked differences in their natural history, physiology, and morphology (e.g., limbless squamates). In recent years, several methodological innovations have been proposed for age assessments of amphibians and reptiles, but traditional methods based on the identification of morphological growth marks still represent the more efficient alternative. This is also due to the lack of testing of the new methods, which leaves their efficiency widely unexplored and does not allow for an accurate assessment of their potential on most taxa of amphibians and reptiles. Nevertheless, the future of age estimation of natural populations is probably linked to either the development of new methods or to the multidisciplinary, synergic application of the existing ones.

## Figures and Tables

**Table 1:** Methods for age determination using growth marks applied to the different groups of amphibians and reptiles as classified by traditional (Linnaean) taxonomy. The number in superscript shows a study example in which the technique has been successfully applied for a certain group. Further studies and taxon-specific range of applicability of different bones/mineralized tissue are reported in the main text. T: tested NA: not applicable; NT: not tested although potentially applicable.

	Scutes Growth Marks 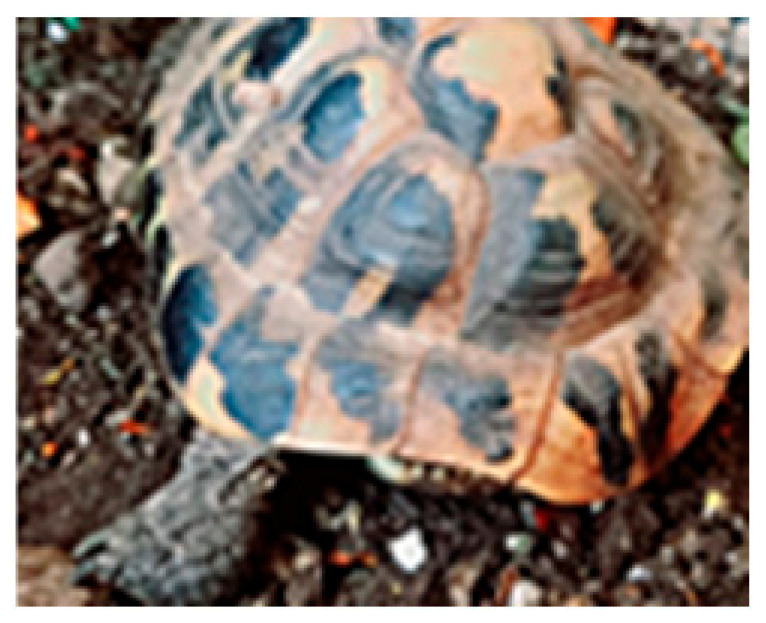	Claws Growth Marks 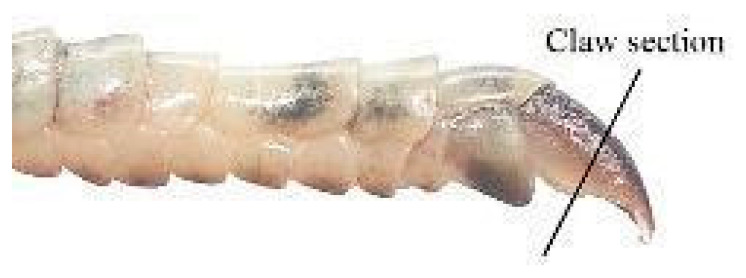	Skeletochronology		
			Cranial Bones 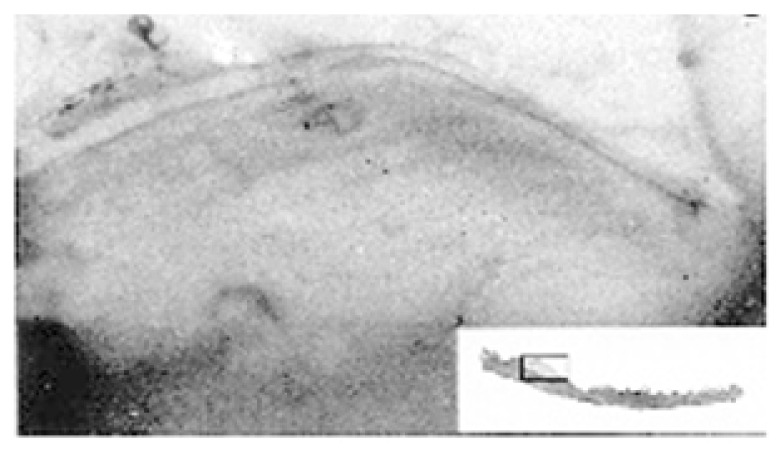	Limb Long Bones 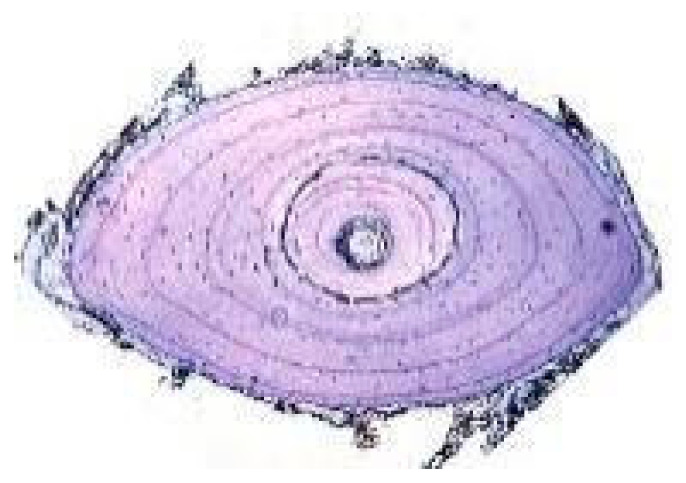	Vertebrae 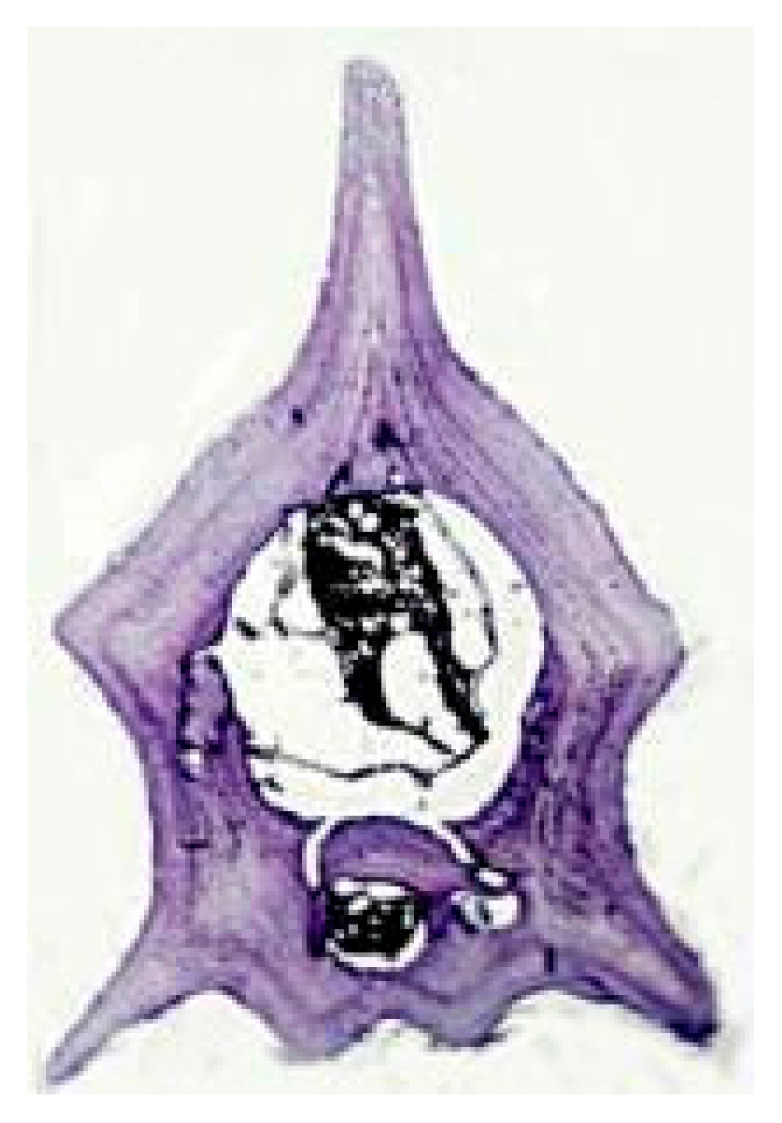
Frogs	NA	NA	T ^4^	T ^9^	NT
Salamanders	NA	NA	T ^5^	T ^10^	NT
Caecilians	NA	NA	NT	NA	T ^15^
Turtles	T ^1^	T ^2^	T ^6^	T ^11^	NT
Crocodiles	NT	NT	T ^7^	T ^12^	NT
Lizards	NT	T ^3^	T ^7^	T ^13^	T ^16^
Amphisbenians	NT	NA	NT	NA	NT
Snakes	NT	NA	T ^8^	NA	T ^17^
Tuataras	NT	NT	NT	T ^14^	NT

^1^ [52]; ^2^ [53]; ^3^ [54]; ^4^ [57]; ^5^ [55]; ^6^ [65]; ^7^ [56]; ^8^ [58]; ^9^ [59]; ^10^ [60]; ^11^ [61]; ^12^ [62]; ^13^ [63]; ^14^ [64]; ^15^ [66]; ^16^ Only for short-limbed [67] or limbless lizards [68]; ^17^ [69].

## Data Availability

Not applicable.
